# UPLC-MS/MS Metabolomics Reveals Babao Dan’s Mechanisms in MASH Treatment with Integrating Network Pharmacology and Molecular Docking

**DOI:** 10.3390/ph18081111

**Published:** 2025-07-25

**Authors:** Shijiao Zhang, Yanding Su, Ao Han, He Qi, Jiade Zhao, Xiangjun Qiu

**Affiliations:** 1 College of Basic Medicine and Forensic Medicine, Henan University of Science and Technology, Luoyang 471023, China; zhangshijiao@stu.haust.edu.cn (S.Z.); tsyd200001@163.com (Y.S.); qihe1125@stu.haust.edu.cn (H.Q.); qqzj123@stu.haust.edu.cn (J.Z.); 2 School of Nursing, Henan University of Science and Technology, Luoyang 471003, China; 15237992753@163.com

**Keywords:** BBD, MASH, network pharmacology, metabolomics, molecular docking

## Abstract

**Background**: Metabolic dysfunction-associated steatohepatitis (MASH) is a progressive disease that easily develops into cirrhosis and hepatocellular carcinoma, but its pathogenesis is not clear, and most therapeutic drugs have obvious limitations. However, Babao Dan (BBD) has a good therapeutic effect on liver disease, but its treatment mechanism is still to be studied. Therefore, we further investigated the mechanism of BBD in treating MASH. **Methods**: We predicted BBD-related targets through network pharmacology and further verified the binding ability of BBD-related targets through molecular docking. We also detected relevant indicators before and after model treatment, as well as metabolomics analysis and identification of the mechanism of action of BBD on MASH. **Results**: Through network pharmacology methods, 158 key cross targets and the top 10 core targets were identified, and it was determined that the PI3K-AKT signaling pathway plays an important regulatory role in the treatment of MASH with BBD. The molecular docking results indicate that the representative compounds quercetin and 17 Beta Estradiol have good binding activity with five core targets. Metabolomics has identified four metabolic biomarkers, such as Piceid, and it is determined that the key pathway for BBD treatment of MASH is the bile secretion pathway. **Conclusions**: BBD effectively treats MASH by modulating Piceid and other biomarkers, targeting ESR1 and other core proteins via quercetin and 17-beta-estradiol, and regulating the PI3K-AKT and bile secretion pathways to alleviate liver injury.

## 1. Introduction

Nonalcoholic steatohepatitis (NASH) is the second stage in the spectrum of nonalcoholic fatty liver disease (NAFLD). As a progressive disease, MASH should be taken seriously in order to prevent further progression to cirrhosis or even hepatocellular carcinoma [1,2]. The pathogenesis of NASH is currently unclear, and basic treatments such as weight loss, dietary control, and exercise are the preferred therapeutic tools for treatment. However, patient compliance is poor, and these methods are not effective in the treatment of NASH and liver fibrosis [3]. While drugs for treating and improving NASH have been developed, they have significant limitations and their effectiveness and safety are still being investigated in clinical studies. Therefore, continuous clinical and preclinical research is needed on existing and potential drugs to improve the treatment of NASH. Recently, experts have suggested using the terms ‘metabolic-associated fatty liver disease’ (MAFLD) and ‘metabolic dysfunction-associated steatohepatitis’ (MASH) to replace NAFLD and NASH in the context of metabolic syndrome [4].

BaBao Dan (BBD) is an imperial medicine with a history of more than 400 years and is often used in modern medicine as an adjunct in the treatment of chronic liver disease. Its uniqueness lies in the presence of a variety of precious natural ingredients such as Calculus Bovis (Niu Huang), Snake Bile (She Dan), Antelope Horn (Ling Yang Jiao), Pearl (Zhen Zhu), Moschus (She Xiang), and Notoginseng Radix et Rhizoma (San Qi) (all plant names were verified through MNPS; http://mpns.kew.org, accessed on 20 January 2025). From the perspective of Chinese medicine, BBD has multiple effects such as reducing temperature, detoxification, calming the liver, reducing wind, relieving pain, and improving eyesight. It can also dilate capillaries, relax the biliary sphincter, dissolve stones, and improve microcirculation, and has anti-inflammatory, choleretic, and antioxidant effects. BBD is widely used clinically in the treatment of many hepatobiliary diseases, with some studies confirming [5] the effectiveness and safety of BBD in improving liver function in patients with jaundiced viral hepatitis. Some studies have also shown [6] that since BBD has no toxic side effects and can significantly improve clinical symptoms and liver function in patients and reduce enzymes and jaundice, it can be used in the treatment of viral hepatitis. These studies provide strong support for the use of BBD in the treatment of viral hepatitis with significant results. Clinical studies have also shown [7] that BBD can promote the recovery of liver detoxification and metabolism in patients after hepatectomy and reduce postoperative damage to hepatocytes; other studies have shown that [8] BBD can reduce the acute inflammatory response caused by various reasons through signaling pathways such as AMFK and cellular autophagy and thus play a role in protecting the liver. BBD was recorded in the Compendium of Materia Medica by Li Shizhen as early as 1596. It has the effects of nourishing the qi and blood, regulating and warming meridians, reducing cold, alleviating wind, and relaxing muscles. BBD was also recorded in the Dictionary of Traditional Chinese Medicine by the China Academy of Chinese Medical Sciences in 1998. Therefore, in this study, we explored BBD’s action in a MASH rat model.

The clinical research results of BBD are consistent with the preclinical research results, and all the experiments mentioned above showed a decreasing trend in AST and ALT after treatment. In addition, the side effects of BBD are very small, and the side effects are mainly gastrointestinal reactions, which are transient and do not cause long-term effects. In terms of drug interactions, if BBD is used in combination with anticoagulants for a long time, it may increase bleeding volume and requires close monitoring of relevant indicators, but it does not have severe interactions with most drugs used to treat liver disease.

MASH is prone to develop and deteriorate into more serious cirrhosis or even hepatocellular carcinoma, and the mechanism of its pathogenesis has not yet been clarified, and BBD has a good therapeutic effect on it, so the present experiments used network pharmacology, metabolomics, and animal-related experimental validation methods to further explore the therapeutic potential of BBD on MASH.

## 2. Results

### 2.1. Network Pharmacology

#### 2.1.1. BBD and MASH Common Target Identification

**Figure 1:**
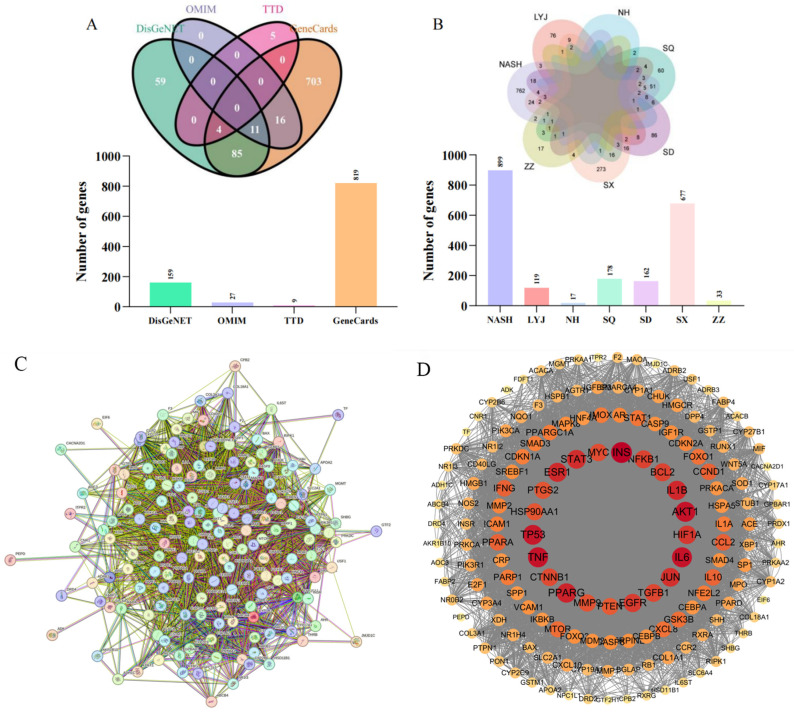

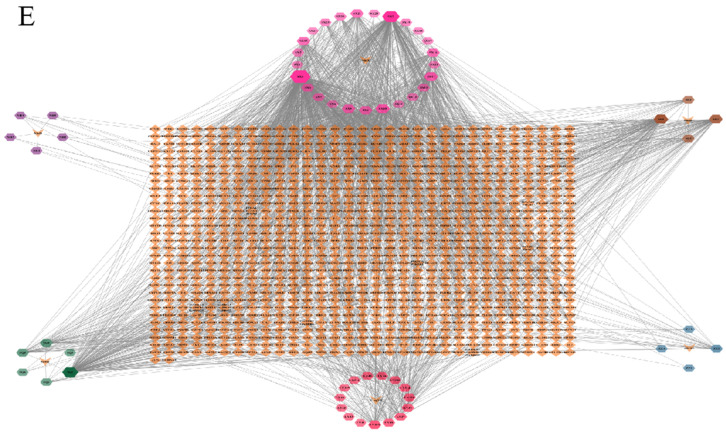

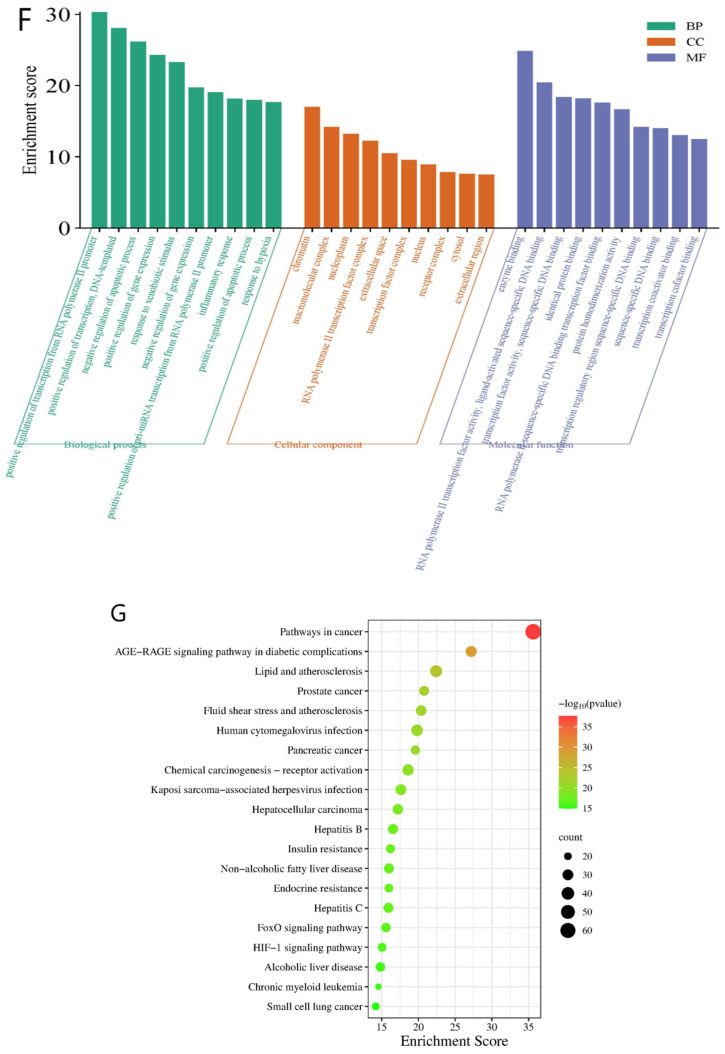
(**A**) Intersection Venn diagram and corresponding bar chart of four disease databases. (**B**) intersection Venn diagram and corresponding bar chart of six known components of MASH and BBD. (**C**) PPI image obtained from STRING. (**D**) PPI image visualized by Cytoscape 3.9.1. (**E**) Visualization of drug active ingredient target map. (**F**) Visualization of drug active ingredient target map. (**G**) Visualization of drug active ingredient target map.

There are six major active components known to be present in BBD that play key roles in the drug’s mechanism of action. In order to gain a more comprehensive understanding of the pharmacological effects of BBD, 137 different bioactive compounds (Table 1) were identified after a series of rigorous screenings (mainly screened in TCMSP database with OB ≥ 30%, DL ≥ 0.18). In addition, we also systematically integrated and analyzed target information from various databases and obtained 963 unique BBD targets (Figure 1A) and 899 targets related to MASH (e.g., Figure 1B), with a total of 158 intersecting targets.

#### 2.1.2. PPI Network and Core Targets

After using STRING to obtain the protein interaction network graph, the downloaded TSV format file was imported into Cytoscape 3.9.1 to obtain 158 nodes and 7354 edges. After selecting Analyze Network, the gene target size, font size, transparency, and color shades were set as the standards to draw the PPI plot (Figure 1C,D). In Centiscape2.2, Betweenness > 122.341772, Closeness > 0.003668, and Degree > 46.544303 were selected as the parameter thresholds after topology with the above three metrics, and then the top 10 core targets were obtained after screening (Table 2).

#### 2.1.3. Drug Active Ingredient Target Map

After importing Cytoscape 3.9.1, 1028 nodes and 2059 edges were obtained. The orange “V” shapes represent the six active pharmaceutical ingredients, and the active pharmaceutical ingredients are surrounded by their corresponding active compounds, with the larger ones indicating the highest correlation, i.e., degree value. Examples include SX5 (17-beta-estradiol), SX2 testosterone, SD4 taurocholic acid, ZZ2 copper, LYJ13 tryptophan, and SQ2 quercetin. The relationship between the three is visualized in Figure 1E.

#### 2.1.4. Enrichment Analysis

After visualization, as can be seen in Figure 1F, the targets of BBD for the treatment of MASH are mainly enriched for the positive regulation of transcription from RNA polymerase II promoter (GO:0045944), the positive regulation of transcription, biological processes such as DNA templates (GO:0045893), cellular components such as chromatin (GO:0000785), macromolecular complexes (GO:0032991), enzyme binding (GO:0019899), RNA polymerase II transcription factor activity, ligand-activated sequence-specific DNA binding (GO:0004879), and other molecular functions.

Using KEGG pathway analysis, we further explored the functions and signaling pathways of BBD anti-MASH targets. The results showed that a total of 178 statistically significant BBD-MASH-related pathways were identified. Among them, the top 20 pathways with the highest enrichment significance are presented as significantly enriched bubble plots in Figure 1G. Notably, the pathways with the smallest *p*-values were pathways in cancer. In addition, the nonalcoholic fatty liver disease pathway may be an important BBD-MASH therapeutic pathway in this study. Both pathways involve a common signaling mechanism, the PI3K-AKT signaling pathway (RNO04151).

### 2.2. Molecular Docking

**Figure 2:**
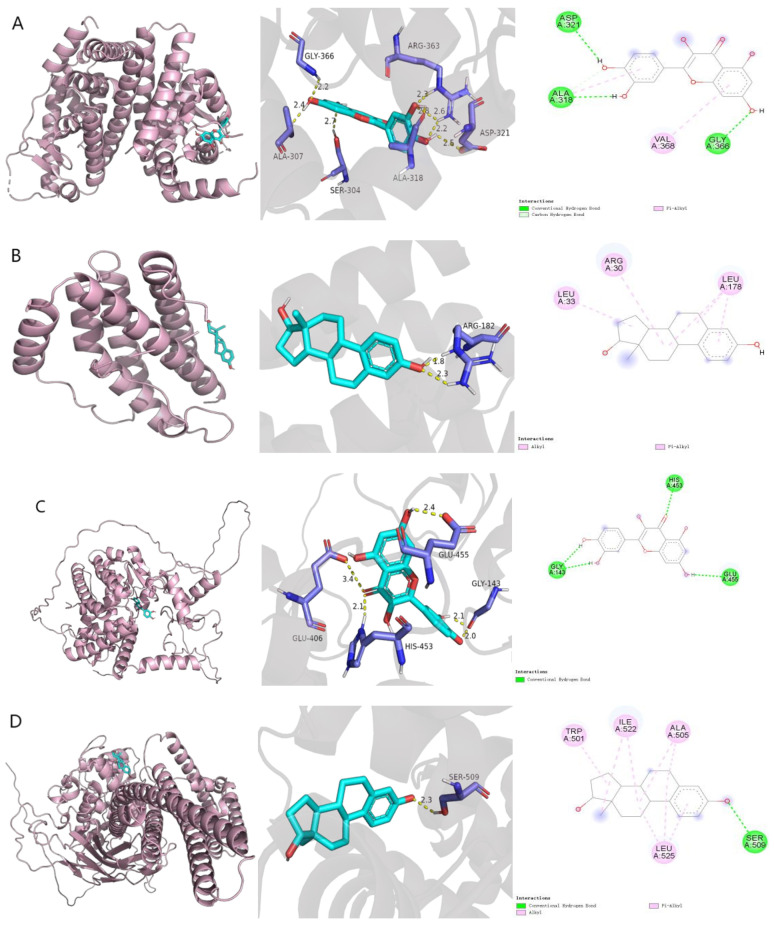

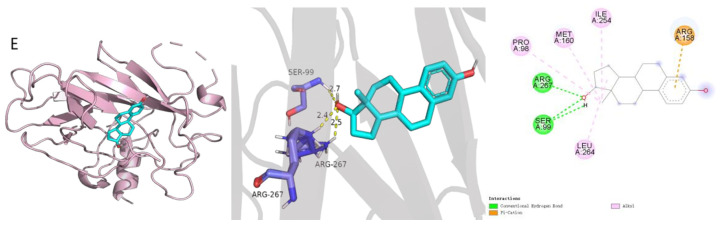
(**A**) ESR1 with quercetin; (**B**) IL6 with 17-beta-estradiol; (**C**) PPARG with quercetin; (**D**) STAT3 with 17-beta-estradiol; (**E**) TP53 with 17-beta-estradiol. Blue represents receptors, cyan represents ligands, and red represents the main interaction sites between receptor ligands.

After docking the top 10 core target proteins with their corresponding components (Table 3), it was found that those with a binding energy < −7.0 kcal/mol were ESR1 with quercetin, IL6 with 17-beta-estradiol, PPARG with quercetin, STAT3 with 17-beta-estradiol, and TP53 with 17-beta-estradiol. The results of their molecular docking are shown in Figure 2, with TP53 binding most stably to 17-beta-estradiol, mainly through the formation of hydrogen bonds with ARG-267 and SER-99 amino acid residues of 17-beta-estradiol target proteins; hydrophobic interactions with PRO-98, MET-160, ILE-254, and LEU-264; and strong Pi–Cation interactions with ARG-158, which together enhance the stability of the complex. These components can bind well to the active site of the core target and exhibit strong binding activity. We can hypothesize that the two representative compounds in BBD, quercetin and 17-beta-estradiol, may exert their therapeutic effects by binding to the five core target proteins of MASH, including ESR1.

In conclusion, quercetin and 17-beta-estradiol, key compounds found in BBD, significantly impact the pathogenesis and treatment of MASH. Additionally, the signaling pathways and biological processes associated with BBD-MASH are crucial for understanding this condition. Network pharmacology and molecular docking studies offer valuable insights and potential mechanisms for the efficacy of BBD in treating MASH.

### 2.3. Changes in Body Weight and Liver Index in Rats

**Figure 3:**
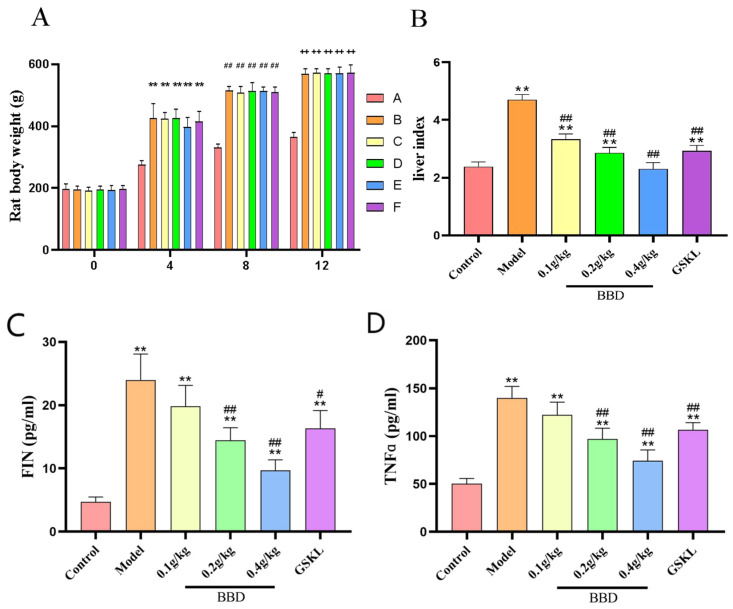

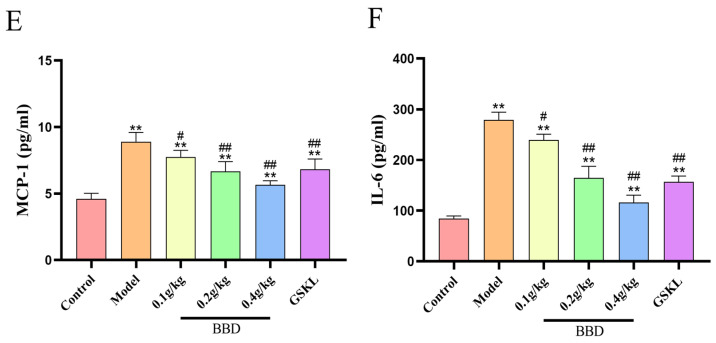
(**A**) Changes in body weight of rats at 0, 4, 8, and 12 weeks. * represents the comparison with week 0, # represents the comparison with week 4, and + represents the comparison with week 8: ** *p* < 0.01, ## *p* < 0.01, ++ *p* < 0.01. (**B**) Liver index chart of each group of rats after treatment. (**C**) FIN level. (**D**) TNFα level. (**E**) MCP-1 level. (**F**) IL-6 level. * represents the comparison with Group A, and # represents the comparison with Group B. ** *p* < 0.01, # *p* < 0.5, ## *p* < 0.01.

The results showed (Figure 3A,B) that the body weight of the rats in the blank control group remained relatively stable throughout the modeling process, while the body weights of rats in the remaining groups gradually increased, and the increase in body weight was obvious.

The liver index (liver weight/rat body weight × 100%) of the rats in the blank control group was relatively low, indicating that the liver was in good condition and not significantly damaged. The hepatic index of the rats in the model group was significantly elevated, indicating that the liver may be damaged or diseased. After treatment, the liver indices in all treatment groups were significantly reduced compared to the model group, indicating effective improvement in liver health. The high-dose BBD group demonstrated the most notable results.

### 2.4. ELISA Results

The results showed (Figure 3C–F) that the serum levels of inflammatory factors as well as the FIN levels changed significantly in all groups of rats after different treatments.

Inflammatory factors (TNFα, MCP-1, IL-6): The levels of each factor were significantly elevated in the model group, indicating that an inflammatory response occurred in the rats. The inflammatory factor levels decreased in all BBD dose groups compared to the model group in a dose-dependent manner, with the most significant reduction observed in the high-dose BBD group. There was also a significant decrease in the GSKL group, but it was slightly less effective than in the high-dose BBD group.

FIN levels: These levels were significantly higher in the model group, indicating that the liver becomes less sensitive to insulin as MASH progresses. The FIN levels decreased in all BBD dose groups compared to the model group, and the higher the dose, the greater the decrease. The FIN levels also decreased in the GSKL group, but the effect was not as pronounced as in the high-dose BBD group.

### 2.5. Biochemical Index Testing Results

**Figure 4:**
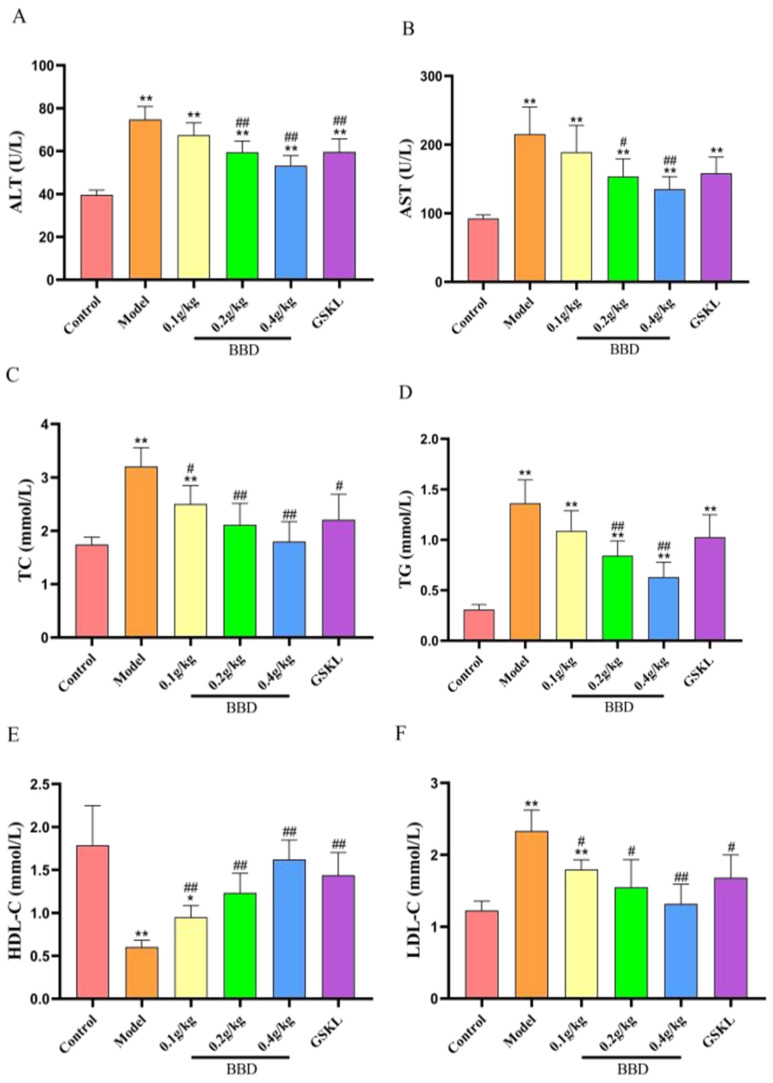

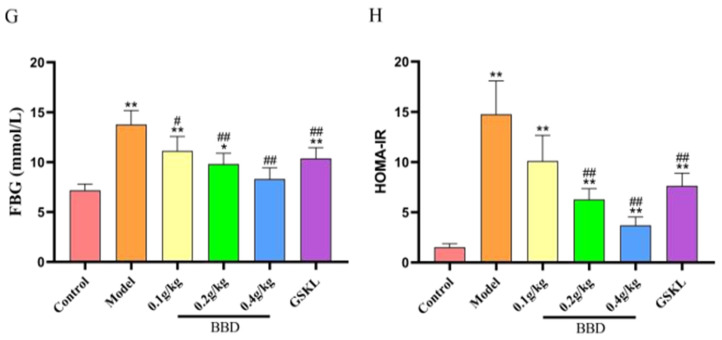
(**A**) ALT level; (**B**) AST level; (**C**) TC level; (**D**) TG level; (**E**) HDL-C level; (**F**) LDL-C level; (**G**) FBG level; (**H**) HOMA-IR. * represents the comparison with Group A, and # represents the comparison with Group B. * *p* < 0.5, ** *p* < 0.01, # *p* < 0.5, ## *p* < 0.01.

The results showed (Figure 4) that significant changes in various biochemical indices in serum as well as insulin resistance index (HOMA-IR) were observable in all groups of rats after the different treatments.

FBG: The FBG levels were significantly higher in the model group, indicating that insulin resistance was induced by modeling in this experiment. The FBG levels decreased in all BBD dose groups compared with the model group in a dose-dependent manner, with the most significant decrease in the high-dose BBD group. There was also a significant decrease in FBG levels in the GSKL group, but it was slightly less effective compared to the high-dose BBD group.

ALT and AST: The ALT and AST levels were significantly elevated in the model group, indicating impaired liver function. These levels decreased in all BBD dose groups compared to the model group, with greater reductions observed at higher doses. The ALT and AST levels also decreased in the GSKL group, but the effect was not as pronounced as in the high-dose BBD group.

Lipid indexes (TC, TG, HDL-C, LDL-C): In the model group, the TC and TG levels were significantly elevated, HDL-C levels were reduced, and LDL-C levels were increased, indicating potential disturbances in lipid metabolism. All BBD dose groups showed varying degrees of improvement in lipid levels, with the high-dose BBD group demonstrating the most notable effects, with significant reductions in TC and TG levels, increased HDL-C levels, and decreased LDL-C levels. The GSKL group also showed some improvement in lipid levels, but the overall effect was less than that of the high-dose BBD group.

HOMA-IR: HOMA-IR was significantly elevated in the model group, indicating severe insulin resistance. All BBD treatment groups exhibited significantly reduced HOMA-IR in a dose-dependent manner, with the greatest decrease observed in the high-dose BBD group. The GSKL group also exhibited reduced HOMA-IR, but the effect was less pronounced than in the high-dose BBD group.

### 2.6. Staining Results

**Figure 5:**
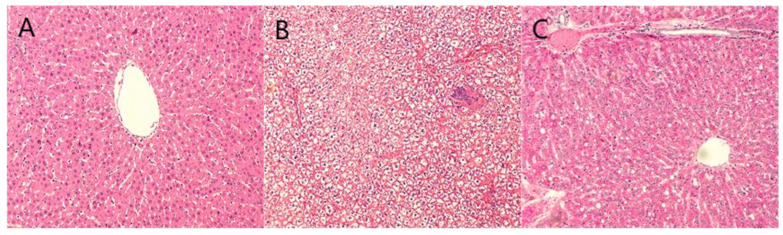

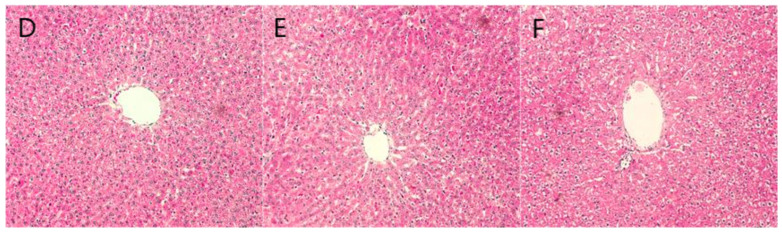
HE staining (**A**) Blank control group; (**B**) model group; (**C**) low-dose BBD treatment group; (**D**) medium-dose BBD treatment group; (**E**) high-dose BBD treatment group; (**F**) GSKL treatment group. (Microscope magnification: 20×).

**Figure 6:**
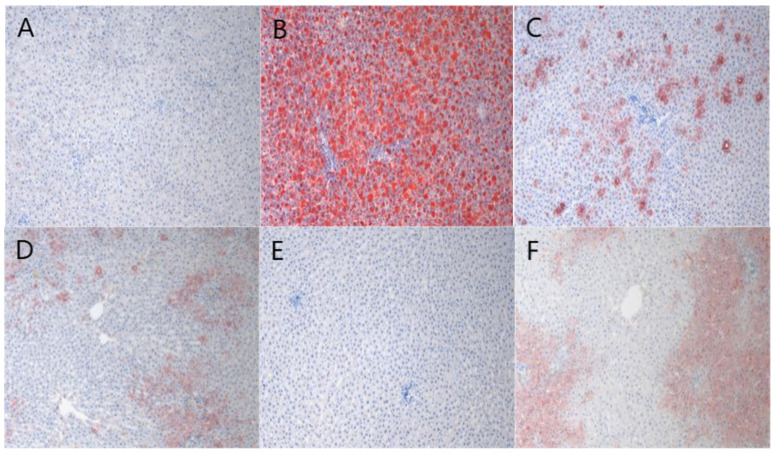
Oil Red O staining (**A**) Blank control group; (**B**) model group; (**C**) low-dose BBD treatment group; (**D**) medium-dose BBD treatment group; (**E**) high-dose BBD treatment group; (**F**) GSKL treatment group. (Microscope magnification: 20×).

**Figure 7:**
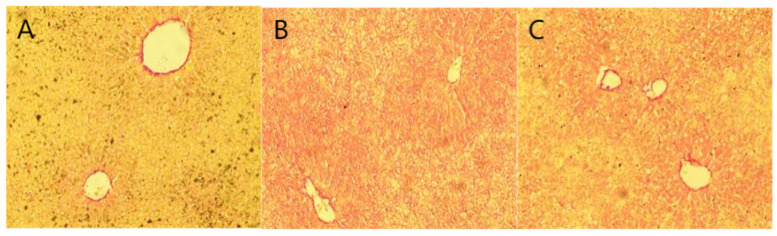

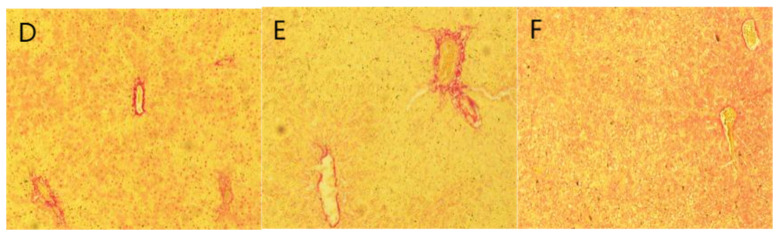
Sirius Red staining (**A**) Blank control group; (**B**) model group; (**C**) low-dose BBD treatment group; (**D**) medium-dose BBD treatment group; (**E**) high-dose BBD treatment group; (**F**) GSKL treatment group. (Microscope magnification: 20×).

The HE staining (Figure 5) results showed that the liver lobule structure of the blank control group remained intact, and liver cells were arranged radially around the central vein. In contrast, the model group exhibited significant lipid droplet aggregation and neutrophil inflammation clusters, and the nuclear morphology was deformed, with some even showing nuclear condensation. In the BBD dose groups and GSKL group, the liver structure gradually tended to be normal, and the degree of steatosis was significantly reduced. The results of Oil Red O staining (Figure 6) showed that the blank control group had fewer lipid droplets in liver cells, and the degree of hepatic steatosis in the rats was lower. The liver tissue of the model group rats was highly steatotic, with lipid droplet vacuoles filling the cytoplasm and a high relative area of lipid droplets. However, in the BBD dose groups and GSKL group, the lipid droplet vacuoles and relative area were significantly reduced. The Sirius Red staining (Figure 7) results showed that the collagen fiber content in the liver of the blank control group was lower and the staining was lighter, mainly distributed in the portal area and around blood vessels, forming a fine mesh structure. In the model group, collagen deposition in the liver tissue increased significantly, the liver lobule structure was disordered, and the relative area of collagen fibers was higher. However, in the BBD dose groups and GSKL group, the degree of collagen deposition was reduced. The above three results all indicate that BBD has a good effect on the treatment of MASH.

### 2.7. Metabolomics

#### 2.7.1. Evaluation of the Quality of the Experiments

Two strategies were used to assess and analyze the systematic stability of the experiments in this project: comparing the mass spectral total ion flow plots of the QC samples, and performing multivariate statistical analyses of the overall samples.

**Figure 8:**
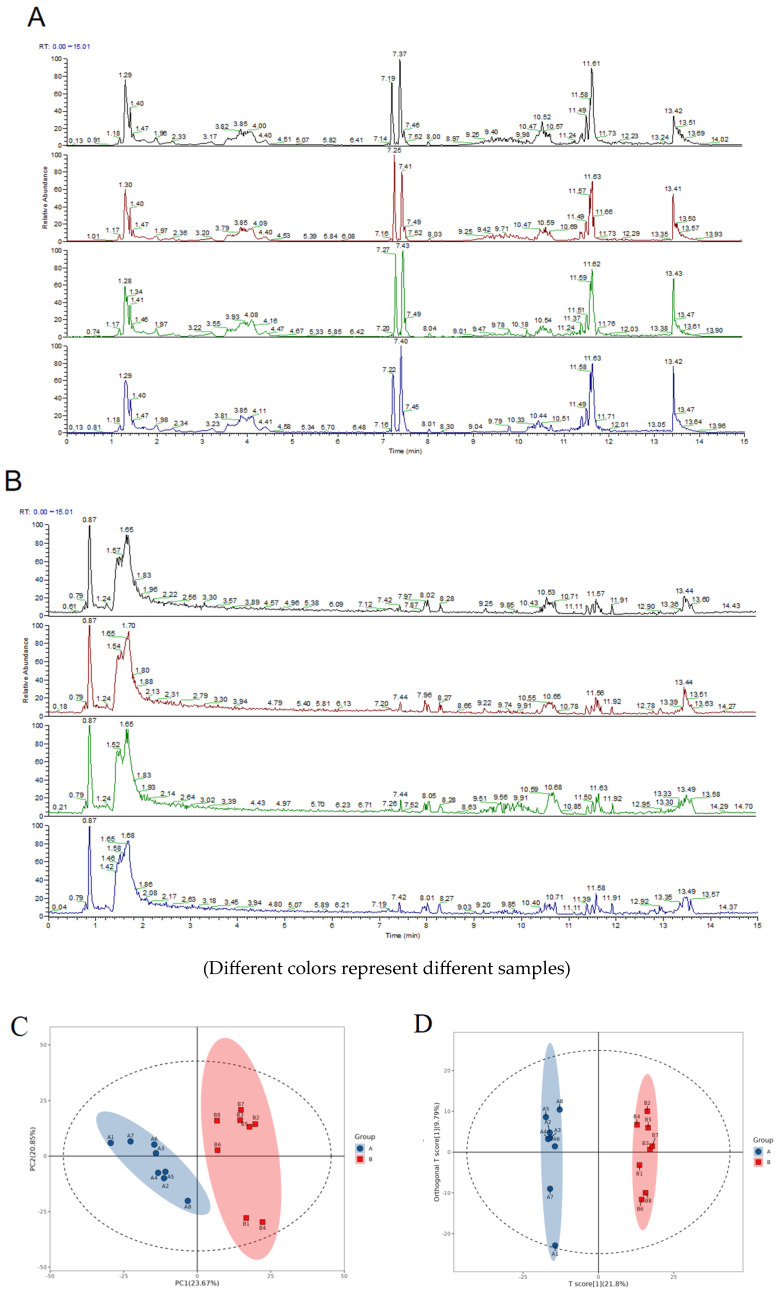

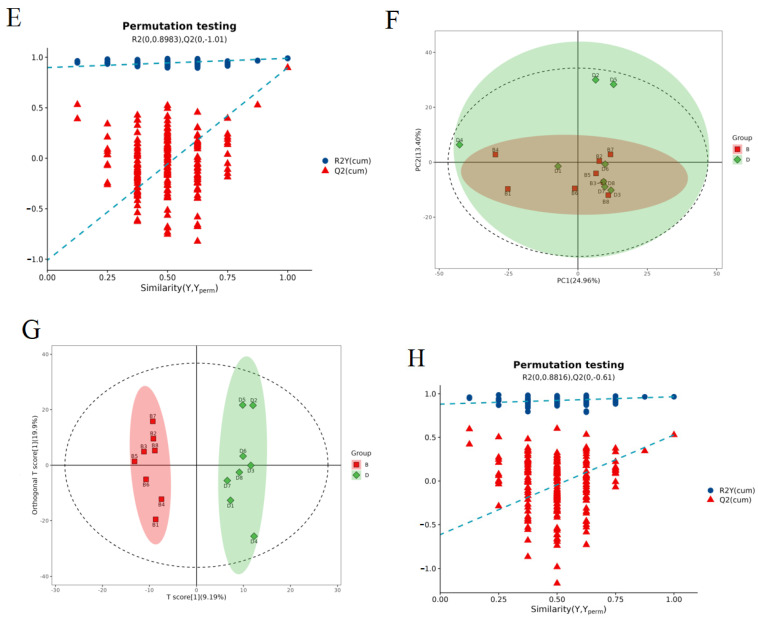
(**A**) Base peak spectrum of QC sample in positive ion mode; (**B**) base peak spectrum of QC sample in negative ion mode; (**C**) PCA score map of comparison groups A and B; (**D**) OPLS-DA score map of comparison groups A and B; (**E**) OPLS-DA permutation test map of comparison groups A and B; (**F**) PCA score map of comparison groups B and D; (**G**) OPLS-DA score map of comparison groups B and D; (**H**) OPLS-DA permutation test map of comparison groups B and D. The blue dots represent the R2Y or Q2Y values of the model after permutation; The red dots represent the true values of the original model. The line passing through the blue dot is the regression line, which is the trend line of the permutation test results, showing the distribution of R2Y/Q2Y with increasing permutation times; The line passing through the red dot is the reference line, which is the value of the original model used to compare the significance of the permutation results.

The peak mass spectra of the QC samples in positive and negative ion detection modes are shown in Figure 8A,B. A comparative analysis showed that the response intensities and retention times of the chromatographic peaks exhibited a high degree of consistency, which fully demonstrated that the variation brought about by the instrumental error was relatively small throughout the whole experimental process, thus ensuring the high reliability of the acquired data.

Multivariate statistical analysis chart: As shown in Figure 8C–H, SIMCA-P 14.1 (Umetrics, Umea, Sweden) was used for the PCA, Partial Least Squares Discriminant Analysis (OPLS-DA), and OPLS-DA permutation tests to evaluate the accuracy of the model. It can be seen that, in each comparison group, the intercept of the Q2 regression line in the OPLS-DA permutation test is less than 0. At the same time, as shown in Table 4, R2Y and Q2 are greater than 0.5 and close to 1. This indicates that the OPLS-DA model established based on the experimental data did not experience overfitting and is very reliable.

#### 2.7.2. Significantly Different Metabolite Statistics

Based on the OPLS-DA model, we used Variable Importance in Projection (VIP) to evaluate the influence and explanatory power of the expression patterns of each metabolite on sample classification and discrimination, in order to discover differential metabolites with practical biological significance. By setting VIP > 1 as the preliminary screening criterion, differential metabolites between groups were identified. Univariate statistical analysis was then used to further confirm whether these metabolites exhibited significant differences between groups. Metabolites selected based on a VIP value > 1 and *p*-value < 0.05 are considered to have significant differences. In addition, FC ≥ 1 indicates the upregulation of metabolites, while an FC value < 1 indicates the downregulation of metabolites.

By integrating data between groups, 417 significantly different metabolites were screened out by us in the positive and negative ion mode when comparing groups A and B, of which 200 metabolite concentrations were upregulated and 217 were downregulated. A total of 118 significantly different metabolites were identified in both positive and negative ion modes in the B vs. D comparisons, with 34 metabolite concentrations upregulated and 84 downregulated. In this experiment, metabolites with VIP > 1.0 and 0.05 < *p* < 0.1, i.e., metabolites with differences, were not examined and therefore were not screened, and we only analyzed and discussed significantly different metabolites.

#### 2.7.3. Differential Metabolite Significance Analysis

**Figure 9:**
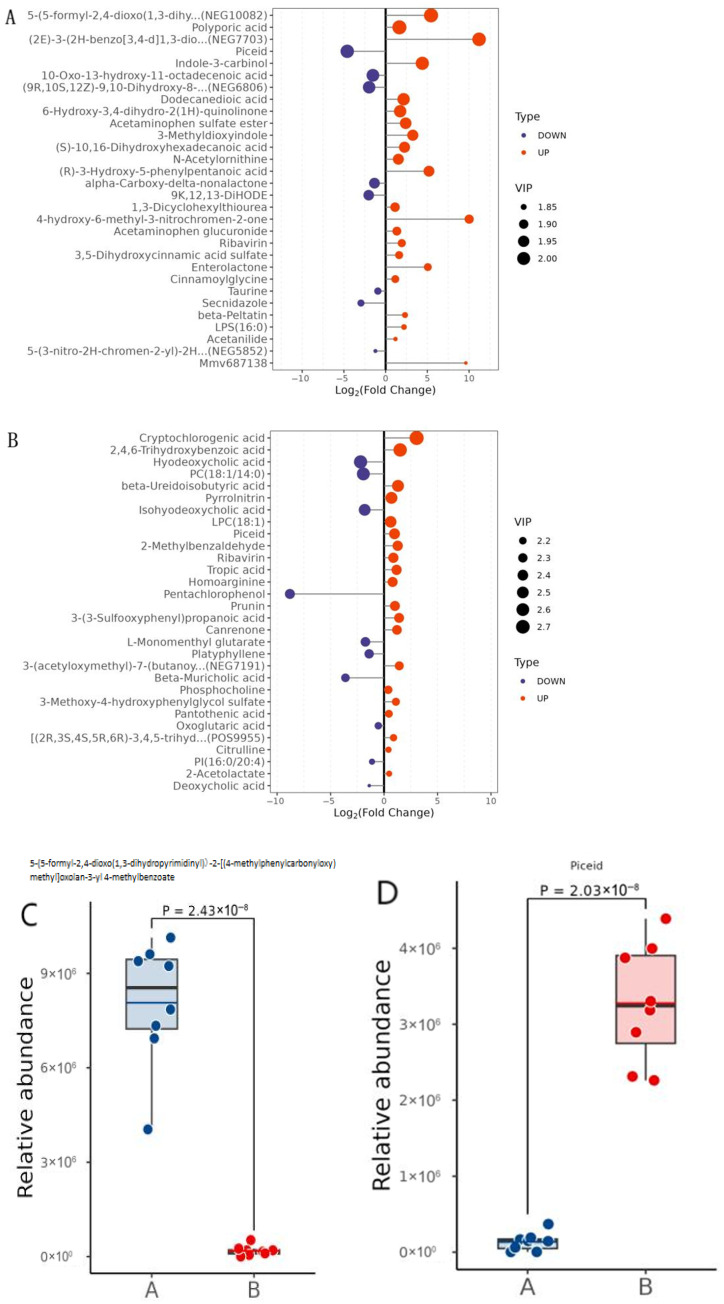

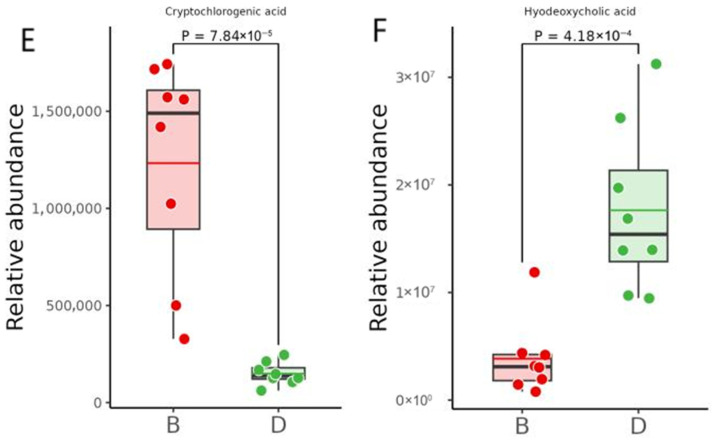
(**A**) Comparison of top 30 differential metabolites and VIP charts for group A and B; (**B**) comparison of top 30 differential metabolites and VIP charts for group B and D; (**C**) shake point plots for NEG10082; (**D**) shake point plots for Piceid; (**E**) shake point plots for cryptochlorogenic acid; (**F**) shake point plots for hyodeoxycholic acid.

Figure 9A,B illustrate some of the comparison group’s differential metabolites of higher importance, with the logarithmic transformation of FC as horizontal coordinates and the metabolites as vertical coordinates. The blue and red dots on the left and right sides represent the downregulated and upregulated differential metabolites, respectively. The size of each dot corresponds to the VIP value: larger dots indicate higher VIP values, signifying greater importance of the variable. As can be seen from the figure, in group A vs. B, 5-(5-formyl-2,4-dioxo(1,3-dihydropyrimidinyl))-2-[(4-methylphenylcarbonyloxy)methyl]oxolan-3-yl 4-methylbenzoate (NEG10082) and Piceid are the most important up- and downregulated significantly different metabolites, respectively; cryptochlorogenic acid and hyodeoxycholic acid (HDCA) are the most important up- and downregulated significantly different metabolites, respectively, in group B vs. D.

#### 2.7.4. Differential Metabolite Abundance Analysis

Figure 9C–F show the average expression of the dithered dot plots of the four significantly different metabolites in the two groups, with the horizontal coordinates being the two groups and the vertical coordinates being the relative expression. The significance in the graph was ascertained using the Student *t*-test. The results showed that the expression of the four metabolites differed significantly between the two groups. By looking at the average expression of each metabolite in the two groups, it was found that NEG10082 was downregulated and Piceid was upregulated in the model group after successful modeling. Cryptochlorogenic acid was downregulated and hyodeoxycholic acid was upregulated after medium-dose BBD treatment.

### 2.8. Metabolomic Bioinformatics Analysis

#### 2.8.1. KEGG

To gain a deeper understanding of the metabolite differences between comparison groups AB, BD, and ABD, we performed KEGG ID mapping and plotted the top 30 most significant KEGG-pathway-enriched bubbles, respectively, thus revealing the function of the metabolites and their interactions more intuitively.

**Figure 10:**
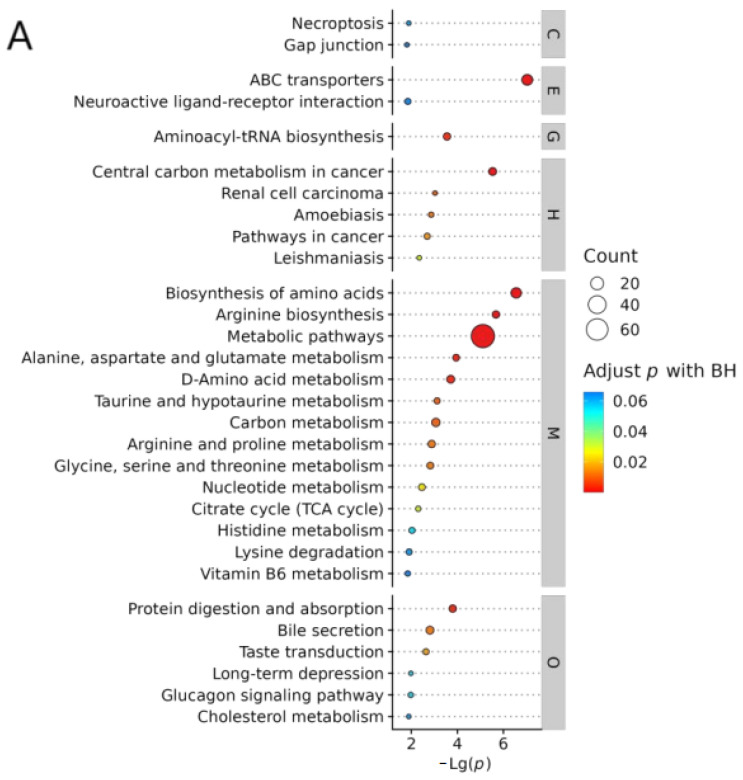

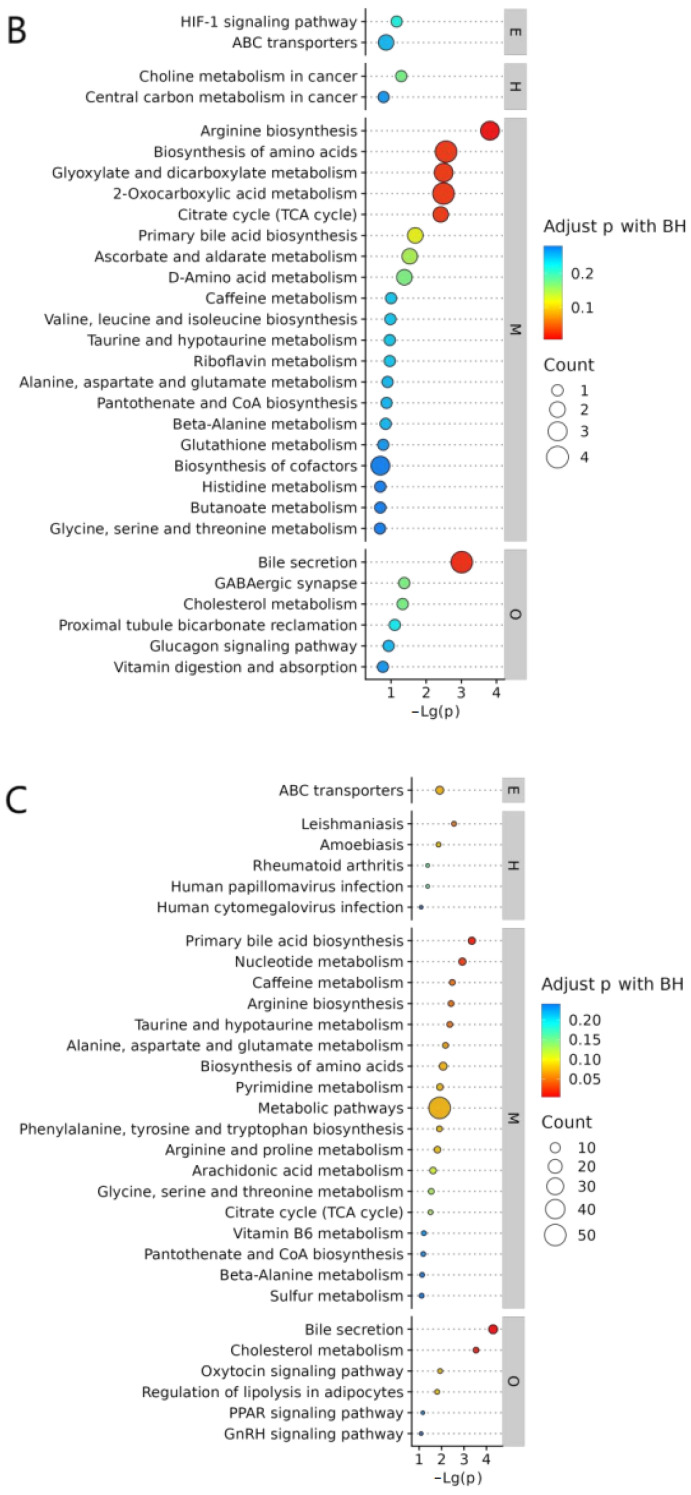

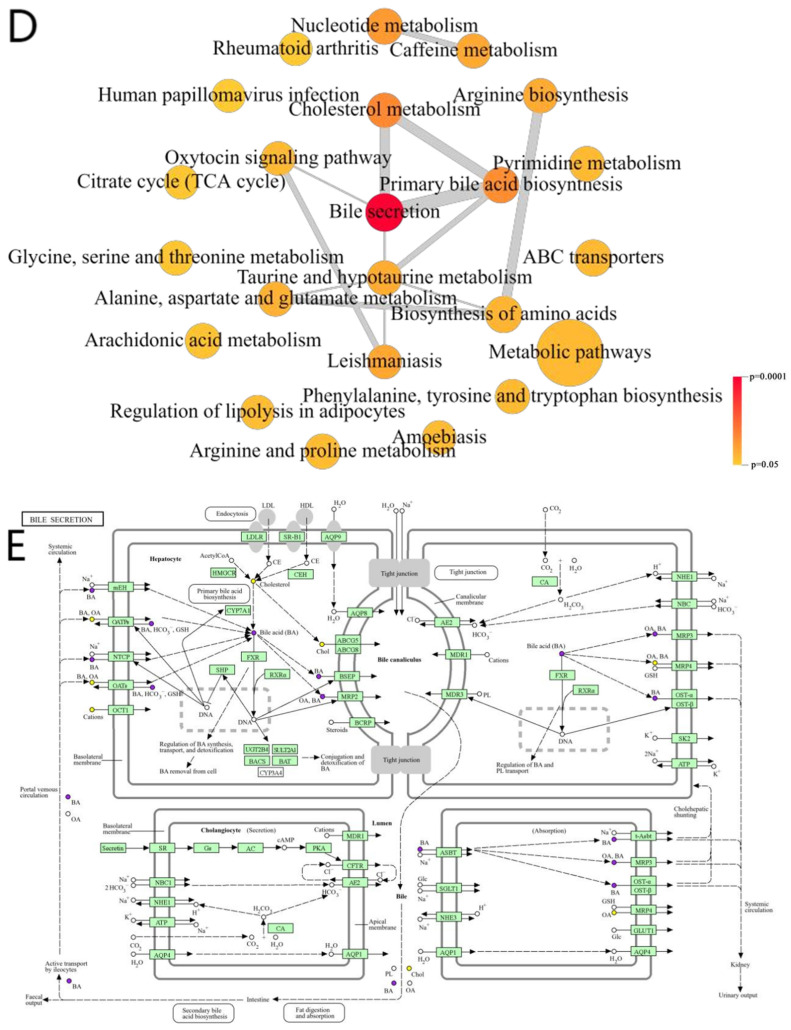

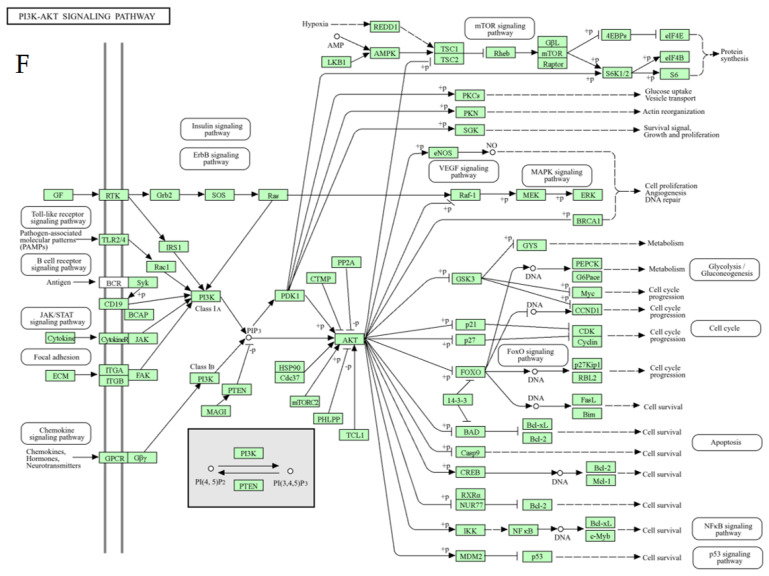
(**A**) Comparison of KEGG enrichment bubble plots in group A vs. B (top 30); (**B**) KEGG enrichment bubble chart of comparison group B vs. D (Top 30); (**C**) comparison of KEGG enrichment bubble charts for group A, B, and D (top 30); (**D**) functional interaction network diagram of pathways; (**E**) KEGG mapping of bile secretion pathway; (**F**) KEGG mapping of the PI3K Akt signaling pathway. M, metabolism; G, genetic information processing; E, environmental information processing; C, cellular processes; O, organismal systems; H, human diseases; D, drug development. The horizontal coordinate represents the negative logarithmic transformation of the *p*-value and the vertical coordinate represents the pathway name. Attributing pathways to the level 1 category, the pathways in each category decreased from top to bottom −log10 (*p*-value), i.e., the *p*-value increased and the significance decreased sequentially. The size of the circle indicates count, i.e., the number of differential metabolites annotated to the pathway; the color of the circle corresponds to the corrected *p*-value, which is more significant from red to blue.

In the AB group studies (Figure 10A), we learned about the direct effects of MASH on metabolites, providing new ideas for treating MASH. Meanwhile, the analysis of the BD group (Figure 10B) demonstrated how BBD specifically affects metabolites in MASH rats. Finally, the ABD group (Figure 10C) study revealed the overall metabolic pathway changes from disease onset to treatment with BBD.

If there is a partial overlap of KEGG pathways in the three comparison groups, it is possible that BBD is a targeted therapeutic pathway for MASH. It can be seen that bile secretion; ABC transporters; biosynthesis of amino acids; arginine biosynthesis; citrate cycle (TCA cycle); cholesterol metabolism; taurine and hypotaurine metabolism; alanine, aspartate, and glutamate metabolism; and central carbon metabolism in cancer are the nine metabolic pathways considered the most important for the treatment of MASH by BBD.

#### 2.8.2. Functional Interaction Network Diagrams for Pathways

As can be seen in Figure 10D, after further functional interaction analysis of the pathways, we found that the bile secretion pathway had the smallest *p*-value, indicating the significance of its relationship with BBD-MASH. Therefore, the bile secretion pathway was regarded as the most important BBD-MASH pathway in this study. Notably, the bile secretion pathway is also closely related to several others, including the cholesterol metabolism pathway and taurine and hypotaurine metabolism.

In the KEGG mapping of the bile secretion pathway (Figure 10E), which involves four main processes—primary bile acid biosynthesis (RNO00120), endocytosis (RNO04144), tight junctions (RNO04530), and fat digestion and absorption (RNO04975)—there are changes in metabolites such as C00486 bilirubin, C00695 bile acids, C03642 taurocholate sulfate, C05122 taurocholate, C01829 thyroxine, and C00187 cholesterol, which may have a role in the treatment of MASH; however, the specific regulatory mechanisms of these metabolites still need to be further studied.

## 3. Discussion

### 3.1. Network Pharmacology

#### 3.1.1. PI3K-AKT Signaling Pathway

In the network pharmacology KEGG pathway analyses, the cancer pathway and the nonalcoholic fatty liver disease pathway were identified as key pathways closely associated with BBD-MASH. In this experiment, we compared the KEGG results from network pharmacology with the KEGG results from the model and medium-dose BBD groups in metabolomics. By comparison, we found a common pathway: the HIF-1 signaling pathway. All three of these pathways involve a common signaling mechanism, the PI3K-Akt signaling pathway (RNO04151, Figure 10F). This finding provides important clues to our in-depth understanding of the pathogenesis and potential therapeutic targets of BBD-MASH.

The PI3K-AKT pathway is a crucial intracellular signaling mechanism that is involved in the regulation of many physiological aspects of cell growth, survival, and metabolic homeostasis [9]. At the same time, it has been shown to be associated with a variety of disorders of glycolipid metabolism metabolic diseases as well as inflammatory responses, affecting systemic metabolic regulation [10].

When the liver is stimulated by unfavorable factors such as a high-fat diet, the PI3K-AKT pathway may be overactivated. This activated state further promotes the accumulation of fat in the liver and exacerbates fatty liver. Further, the activation of this pathway may exacerbate oxidative stress, leading to hepatocyte damage and inflammation. Additionally, the aberrant activation of the PI3K-AKT pathway is closely linked to the progression of liver fibrosis and cirrhosis. Excessive activation of the pathway may promote the proliferation and activation of fibropoietic cells, leading to the accumulation of fibrous tissue in the liver and the subsequent development of cirrhosis [11].

#### 3.1.2. Relationship of ESR1, IL6, PPARG, STAT3, and TP53 to the PI3K-AKT Signaling Pathway

ESR1 plays an important role in breast cancer and other estrogen-related diseases. Its activation can affect signaling in the PI3K-AKT pathway, which regulates cell growth and proliferation [12]. When ESR1 is stimulated, it activates PI3K, which then phosphorylates and activates AKT, promoting cell survival and proliferation.

IL-6 is a multifunctional cytokine that plays a crucial role in immune and inflammatory responses. IL-6 binding to its receptor allows signaling through the downstream JAK-STAT and PI3K-AKT pathways [13]. In particular, the STAT3 signaling pathway, which is considered a major pathway of cancer inflammation, interacts with the PI3K-AKT pathway to regulate tumor cell growth and survival.

PPARG acts as a nuclear receptor involved in the regulation of lipid metabolism and inflammatory responses. The activation of PPARG can affect the activity of the PI3K-AKT pathway, which in turn affects cell growth and differentiation [14].

STAT3 is an important transcription factor involved in the regulation of multiple cell signaling pathways. In cancer, STAT3 activation is usually closely associated with tumor growth, invasion, and metastasis. There is also an interaction between STAT3 and the PI3K-AKT pathway [15], which together regulate cell growth, survival, and metabolic processes.

Finally, TP53 is a well-known tumor suppressor gene that plays a critical role in regulating the cell cycle, apoptosis, and DNA repair. The activation of the PI3K-AKT pathway can negatively regulate the function of p53, thereby inhibiting its oncogenic effects [16]. In contrast, when p53 functions normally, it inhibits the activity of the PI3K-AKT pathway, thereby maintaining normal cell growth and differentiation.

#### 3.1.3. Quercetin and 17-Beta-Estradiol

Quercetin, a natural polyphenolic flavonoid with antioxidant and anti-inflammatory properties, has shown promising results in alleviating MASH by reducing hepatic lipid accumulation, improving mitochondrial function, and enhancing autophagy [17]. Meanwhile, the addition of quercetin to the treatment of MASH helped to reduce the intensity of oxidative stress and enhance the antioxidant protective activity, resulting in a decrease in hepatocyte apoptosis [18].

17-Beta-Estradiol has been shown to enhance the proliferation of cholangiocytes and hepatocytes in vitro, and may delay the onset of liver disease by preventing to some extent the harmful effects of certain potentially toxic bile acids and reactive oxygen species [19]. Moreover, 17-Beta-Estradiol inhibits the action of hepatitis C virus through its intracellular receptor, mainly interfering with the assembly and release of the hepatitis C virus life cycle [20].

### 3.2. Animal Experimentation

#### 3.2.1. GanShuangKeLi

GanShuangKeLi, a hepatoprotective drug commonly used in clinical practice, integrates various precious medicinal herbs, among which Codonopsis Radix (Dang Shen) and Bupleuri Radix (Chai Hu) dominate, and Angelicae Sinensis Radix (Dang Gui), Salviae Miltiorrhizae Radix et Rhizoma (Dan Shen), Atractylodis Macrocephalae Rhizoma (Bai Zhu), and Polygoni Cuspidati Rhizoma (Hu Zhang) are used as adjuncts. In addition, ingredients such as Persicae Semen (Tao Ren), Poria (Fu Ling), Taraxaci Herba (Pu Gong Ying), Prunellae Spica (Xia Ku Cao), Trionycis Carapax (Bie Jia), Aurantii Fructus (Zhi Ke), and Paeoniae Radix Alba (Bai Shao) are added. This unique combination effectively reduces inflammation and liver fibrosis and protects liver cells, thanks to the Chai Hu Saponin contained in Chai Hu and the flavonoids in Codonopsis. In addition, Dan Shen plays an equally important role by promoting the liver uptake and breakdown of specific substances, such as laminin and hyaluronic acid, which, in turn, positively affects liver fibrosis [21,22].

GanShuangKeLi has the effects of soothing the liver and spleen, reducing temperature, protecting the liver, and softening and reducing lumps. It not only significantly enhances the effectiveness of drugs, but also greatly reduces potential side effects and alleviates damage to organs such as the liver and kidneys. Because it is widely applicable for the treatment of hepatitis, liver fibrosis, and liver function damage, we chose Ganshuang granules as the positive control drug in this study.

#### 3.2.2. Indicators

In the present study, the body mass of the MASH rats increased over time due to the administration of special diets, suggesting that a prolonged diet of high-calorie or high-fat foods directly causes obesity and changes in energy metabolism, fat metabolism, and glucose metabolism.

Inflammation is a central pathologic process in the MASH disease process that exacerbates disease progression and may lead to more severe liver injury. Inflammatory factors [23], such as TNFα, IL-6, and MCP-1, play a key role in the pathogenesis of MASH. Elevated levels of these factors exacerbate the inflammatory response in the liver, leading to liver cell damage and fibrosis. The improvement in inflammatory factor results after BBD treatment implies that the drug may attenuate the inflammatory response in the liver of MASH rats by modulating the immune response and inhibiting the production or release of inflammatory factors. This not only helps to relieve the current symptoms of inflammation, but may also stop or slow the progression of the disease and prevent further liver damage.

Insulin resistance is an important pathological feature of MASH [24] and a key factor in metabolic syndrome and type 2 diabetes. When the body’s cellular response to insulin is impaired, higher levels of insulin are required to maintain normal blood glucose levels, often resulting in elevated fasting blood glucose. The improvement in HOMA-IR after BBD treatment may mean that the drug enhances cellular sensitivity to insulin, thereby reducing fasting blood glucose levels. In addition, improving insulin resistance may also have a positive impact on other aspects of MASH, such as reducing fat accumulation and attenuating the inflammatory response, all of which can help improve the pathology of MASH.

After treatment with different doses of BBD and GSKL, hepatic index, hepatic pathology levels, biochemical index levels, and inflammatory factor levels recovered. Among them, the hepatic indices showed better improvement after treatment, indicating the effectiveness of drug treatment on MASH rats, the recovery of liver function, and the reversal of the disease process. Then, three staining methods were used to observe the level of liver pathology in MASH rats, and the results showed that steatosis, hepatic fibrosis, hepatic histological changes, and damage and inflammatory responses were significantly improved in MASH rats after treatment.

In conclusion, the animal experiments in this study support the use of BBD in traditional Chinese medicine. It demonstrates efficacy in reducing temperature and removing toxins, activating blood circulation, and alleviating blood stasis. These effects may positively influence lipid metabolism, reduce fibrosis, and mitigate the inflammatory response in the liver. Meanwhile, BBD was able to significantly improve various indices in MASH rats in a dose-dependent manner. The high-dose group demonstrated the superior therapeutic efficacy of BBD on most measures compared to the positive drug control group.

### 3.3. Metabolomics

#### 3.3.1. Significantly Different Metabolites

In this study, Piceid was upregulated and NEG10082 was downregulated in the model group after successful modeling. This suggests that, when MASH occurs, NEG10082 is downregulated and the biosynthetic pathway associated with this metabolite is inhibited or regulated, which may be due to a reduction in the upstream substrate, a decrease in the activity of the associated enzyme, or other regulatory mechanisms. The upregulation of Piceid may imply that it plays some protective or mitigating role in the pathogenesis of MASH. Combining these two metabolite changes, we can hypothesize that the upregulation of Piceid may be a protective response that contributes to the alleviation of the pathological process of MASH. Meanwhile, it has been shown [25] that Piceid improves MASH, and the downregulation of NEG10082 may reflect an improvement of the condition or adjustment of related metabolic pathways.

Cryptochlorogenic acid was downregulated and hyodeoxycholic acid was upregulated after medium-dose BBD treatment, suggesting that cryptochlorogenic acid may be a negative factor in the pathogenesis of MASH; its downregulation may imply that BBD treatment helps to ameliorate or alleviate the pathology of the liver. It is also possible that some components of BBD directly or indirectly affect the metabolism of cryptochlorogenic acid, leading to its downregulation, which may be an important mechanism for the treatment of MASH with BBD. As a bile acid, the upregulation of hyodeoxycholic acid may enhance the digestion and absorption capacity of fat, which helps to improve the common fat metabolism disorders in MASH. At the same time, by promoting the metabolism of cholesterol and the recycling of bile acids, it may help to lower the levels of cholesterol and triglycerides in the blood, thus regulating lipid balance, which is positive for the treatment of MASH. It has been shown [26] that hyodeoxycholic acid treatment alleviates MAFLD by inhibiting intestinal farnesol X receptor (FXR) and upregulating hepatic CYP7B1. Taken together, the changes in these two metabolites allow us to hypothesize that the BBD treatment of MASH may work through multiple pathways: on the one hand, liver pathology is ameliorated by decreasing the level of cryptochlorogenic acid; on the other hand, fat metabolism and lipid homeostasis are regulated by increasing the level of hyodeoxycholic acid. These two effects may work together to promote therapeutic efficacy in MASH.

Overall, this study identified metabolite changes associated with the pathogenesis and treatment of MASH, providing important clues for a deeper understanding of the pathogenesis of MASH and the search for new therapeutic approaches.

#### 3.3.2. Bile Secretion Pathway

In the present study, the bile secretion pathway was considered to be the metabolic pathway of greatest interest for BDD treatment as it plays a key role in the pathogenesis of MASH. Bile secretion and excretion are essential for fat metabolism, cholesterol homeostasis, and liver detoxification. In the MASH state, abnormalities in the bile secretion pathway may lead to the accumulation of fat in the liver and aggravate liver injury. The four processes associated with it and the changes in multiple metabolites shed light on the complex mechanisms and pathways that may be involved in MASH treatment.

First, it can be assumed that primary bile acid biosynthesis is one of the central processes of the bile secretion pathway. Bile acids [27], such as cholic acid and taurocholic acid, play a pivotal role in fat digestion and absorption as key components of bile. Changes in these metabolites may be a reflection of the regulation of bile acid metabolism during MASH treatment, which, in turn, affects fat metabolism and clearance. Key enzymes such as CYP7A1, CYP8B1, and CYP27A1 play important roles in maintaining bile acid homeostasis. Cholesterol is converted to 7α-hydroxycholesterol catalyzed by CYP7A1, which is subsequently further catalyzed by CYP8B1 to produce bile acids [28]. More than 75% of the bile acids in the human body are produced through this classical pathway. The action of these enzymes ensures the normal synthesis and metabolism of bile acids, which is important for maintaining the physiological balance of the body. Finally, the primary bile acids combine with glycine or taurine to form taurocholic acid or glycocholic acid. Subsequently, these bile acids are secreted into the bile with the assistance of transport mechanisms such as bile salt efflux pumps and multidrug-resistance-associated proteins [29]. This series of biosynthetic and transport processes ensures the correct composition and concentration of bile acids in the bile, thus maintaining the normal physiological function of bile.

Second, changes in endocytosis and tight junctions may be related to the function of hepatocytes and cholangiocytes. Endocytosis influences the cellular uptake and transport of substances, while tight junctions maintain the integrity of the biliary system. Changes in these processes may help to regulate bile secretion and excretion, thereby ameliorating the abnormal biliary function in MASH. In addition, changes in fat digestion and absorption are directly linked to the pathologic process of MASH. MASH is often accompanied by disorders of fat metabolism, and the buildup of fat in the liver can exacerbate liver damage. Therefore, the improvement of fat digestion and absorption processes may be an important part of MASH treatment.

Finally, changes in the metabolism of thyroxine and cholesterol are also of interest. Thyroxine plays a key role in lipid metabolism, and cholesterol is an important component of bile acids and cell membranes. Changes in these metabolites may reflect the overall regulation of lipid metabolism in MASH therapy. One study found that [30] thyroxine receptor agonists showed positive results in the treatment of MASH. Thyroxine or its analogs have the potential to be new targets for MAFLD treatment, showing potential in the treatment of MASH.

#### 3.3.3. Relationship of ESR1, IL6, PPARG, STAT3, and TP53 to the Bile Secretion Pathway

ESR1 plays an important regulatory role in the bile secretion pathway [31]. Estrogen is able to influence bile secretion and excretion processes by binding ESR1. The activation of ESR1 regulates the expression of bile acid synthase, which, in turn, affects bile acid production and secretion. In addition, ESR1 may be involved in the proliferation and differentiation of biliary epithelial cells, thereby maintaining the normal function of the biliary system.

IL-6 plays a crucial role in the inflammatory response. In the occurrence of biliary-related diseases [32], the expression level of IL6 may be elevated, affecting the normal function of the bile secretion pathway by activating downstream signaling pathways.

In the bile secretion pathway, PPARG may influence bile composition and secretion by regulating the expression of genes involved in cholesterol metabolism and bile acid synthesis. In addition, PPARG may be involved in the metabolic regulation of biliary epithelial cells to maintain the homeostasis of the biliary system [33].

In the biliary system, the activation of STAT3 may affect the normal function of the bile secretion pathway by regulating processes such as the proliferation, differentiation, and apoptosis of biliary epithelial cells. In addition, an important role of STAT3 in bile-reflux-associated molecular oncogenic events has been demonstrated [34].

Although TP53 is primarily focused on intracellular signaling regulation, the aberrant expression of TP53 in biliary epithelial cells may also indirectly affect the bile secretion pathway. If TP53 is mutated or inactivated, it may lead to uncontrolled cell proliferation and biliary tumorigenesis, which, in turn, interferes with normal bile secretion and excretion [35].

In conclusion, there are complex and diverse relationships between ESR1, IL6, PPARG, STAT3, and TP53 and the bile secretion pathway. These molecules may work together to maintain the normal function of the biliary system by regulating the processes of proliferation, differentiation, and apoptosis in biliary epithelial cells, as well as the synthesis and secretion of bile components. They may act synergistically through multiple pathways to improve the pathology of MASH.

## 4. Materials and Methods

### 4.1. Chemicals and Reagents

Acetonitriles (LOT: 1.00030.4008) and methanol (LOT:1.06007.4008) were purchased from Merck (Darmstadt, Germany). Formic acid (LOT: 111670) was obtained from Millipore (Burlington, VT, USA).

Babao Dan (LOT: 210603) was purchased from Xiamen Traditional Chinese Medicine Factory Co. (Xiamen, Fujian, China). GanShuangKeLi (LOT: 220523) was purchased from Shandong Buchang Pharmaceutical Co. (Heze, Shandong, China). MASH modeling specific feed (LOT: 20221008) was purchased from Jiangsu Collaborative Medical Biotechnology Co. (Yangzhou, Jiangsu, China). Ordinary feed (LOT: 22083241) was purchased from Beijing Ke’ao Cooperative Feed Company (Beijing, China). The rat fasting insulin (FINS) enzyme-linked immunosorbent assay kit (LOT: Apr 2023), rat tumor necrosis factor alpha (TNFα) enzyme-linked immunosorbent assay kit (LOT: Apr 2023), rat monocyte chemoattractant protein-1 (MCP-1) enzyme-linked immunosorbent assay kit (LOT: Apr 2023), and rat interleukin-6 (IL-6) enzyme-linked immunosorbent assay kit (LOT: Apr 2023) were purchased from Xiamen Lunchangshuo Biotechnology Co. (Xiamen, Fujian, China)

### 4.2. Preparation Process, Quality Control Data, and Chemical Fingerprint of BBD

The Babao Dan used in the study were standardized preparation, it was supplied by Xiamen Traditional Chinese Medicine Factory Co., Ltd. Six Chinese herbal ingredients were used, namely 1 g Calculus Bovis, 1 g Snake Bile, 3 g Antelope Horn, 3 g Pearl, 0.5 g Moschus, and 6 g Notoginseng Radix et Rhizoma, in a ratio of 1:1:3:0.5:6; the extraction method involved slicing or grinding these ingredients into a powder. The powder was then added to an appropriate amount of water and soaked for 24 h. The soaking solution was then heated to boiling and boiled continuously for 1 h, after which the extraction solution was filtered and the filtrate was collected. The filtrate was concentrated to the desired volume during preparation, with an appropriate amount of honey or other sweetener added for seasoning. The concentrate was then made into pills or capsules, and finally BBD was obtained. It is stable, highly safe, and highly reliable. Its chemical fingerprint (HPLC) can be seen in Supplementary Material File S1.

### 4.3. Network Pharmacology

Network pharmacology analysis began with the collection of active compounds from Ling Yang Jiao (LYJ), She Dan (SD), She Xiang (SX), Zhen Zhu (ZZ), Niu Huang (NH), and San Qi (SQ) using the TCMSP, SwissTargetPrediction, and BATMAN-TCM databases, followed by screening based on OB ≥ 30%, DL ≥ 0.18, and Lipinski’s rule of five [36]. Targets were filtered (BATMAN score > 25, SwissTargetPrediction Probability > 0), standardized via UniProt, and visualized using Wayne diagrams. MASH-related targets were mined from TTD, Genecards, OMIM, and DisGeNet using keywords like “nonalcoholic steatohepatitis” and “MASH”, with results visualized in R4.0.3. The intersection targets of BBD and MASH were analyzed in the STRING database (Homo sapiens, interaction score ≥ 0.40), and the PPI network was constructed and visualized in Cytoscape 3.9.1, with core targets identified using the Centiscape2.2 plugin (Betweenness, Closeness, Degree). A “drug-active ingredient-target” network was built in Cytoscape to elucidate BBD’s potential mechanism against MASH. Finally, GO functional annotation and KEGG pathway enrichment were performed using DAVID, with the top 10 GO terms (BP, MF, CC) and top 20 KEGG pathways selected and visualized to clarify the biological processes and metabolic pathways involved.

### 4.4. Molecular Docking

The top 10 core targets were selected for molecular docking with corresponding compounds to verify their affinity. It is generally believed that a binding energy < −4.25 kcal/mol, −5.0 kcal/mol, or −7.0 kcal/mol indicates a certain, good, or strong binding activity, respectively, between the ligand and the receptor. In this study, a binding energy < −7.0 kcal/mol was used as a screening criterion. To obtain the crystal structure of each protein, the PDB database (https://www.rcsb.org/ (accessed on 1 June 2023)) was accessed and the search criteria were followed: “Homo sapiens” and refinement resolution < 3.0. Next, we made necessary preparations for the molecular docking task using AutoDockTools 1.5.6 software to ensure the accuracy and rationality of the protein structure. Meanwhile, the structure of the ligand was optimized to be in a low-energy stable conformation, which helped to more accurately simulate its binding process with the target protein. Next, molecular docking operations were performed using the vina algorithm built into the PyRx 0.8 software. Finally, the results were visualized.

### 4.5. Selection and Grouping of Experimental Animals

Sixty male Sprague-Dawley (SD) rats with a body mass of 180 ± 20 g were selected and provided by the Laboratory Animal Center of Henan University of Science and Technology [Quality Certificate: SCXK (Hubei) 2019-0002]. This study was approved by the Animal Laboratory of Henan University of Science and Technology (202210001). The feeding conditions were as follows: 12 h light/dark cycle (light on, 8:00–20:00); temperature, 22 ± 2 °C; humidity, 50 ± 10%. Food and water were forbidden for 24 h before the experiment, and the whole process of animal experiments followed the Guidelines for Ethical Review of Laboratory Animal Welfare (GB/T35892-2018) [37].

The animals were randomly divided into a blank control group (group A), model group (group B), low-dose BBD group (group C) (0.1 g/kg), medium-dose BBD group (group D) (0.2 g/kg), high-dose BBD group (group E) (0.4 g/kg), and positive drug control group (GanShuangKeLi, GSKL group) (0.9 g/kg), with a total of 6 groups (10 animals in each group) (each dose is calculated based on the equivalent dose ratio between experimental animals and humans, which means that the required dose per kilogram of rat is about 6 times that of humans; the dose calculated by BBD is the medium dose, and the low dose and high dose are reduced or doubling; the dose converted by GSKL is directly recorded as the dose used in the positive drug control group). After passing the acclimatization and feeding phase, modeling was performed based on a weekly intake of a high-fat diet (82.5% regular feed, 2% cholesterol, 10% lard, 5% egg yolk powder, and 0.5% sodium cholate) at 10% of the total body weight of each group, except the blank control group, for 12 weeks.

At the end of the 12th week, blood was collected from the tail vein of each group of rats, immediately frozen in liquid nitrogen, and stored at −80 °C for subsequent analysis. Then, the feed of the other groups was replaced with the same normal feed as the blank control group, and the gavage treatment was started for a period of 1 month. The blank control and model groups were given 10 mL/kg saline; the low-, medium-, and high-dose BBD groups were given 0.1 g/kg, 0.2 g/kg, and 0.4 g/kg BBD suspension, respectively; and the GanShuangKeLi (GSKL) group was given 0.9 g/kg aqueous solution of GSKL. At the end of treatment, blood was again collected from the tail vein of the rats in each group for subsequent ELISA testing as well as the analysis of biochemical indices in order to observe the effects on inflammatory factors and serum biochemical indices before and after treatment with BBD and GSKL. Meanwhile, the histopathological morphology, steatosis, and collagen fiber deposition of the rat livers were observed via HE, Oil Red O, and Sirius Red staining to assess whether the diagnostic criteria for MASH were met. Finally, venous blood was collected from the blank control group, the model group, and the medium-dose BBD group for subsequent metabolomics analysis. At the end of the animal experiments, we euthanized the rats. We deeply anesthetized the rats by intraperitoneal injection of sodium pentobarbital (50 mg/kg); then, we ensured the death of the rats by cardiac perfusion with 4% paraformaldehyde, and finally, we observed respiratory arrest, cardiac arrest, and corneal reflexes in the rats, and all the steps were independently verified by two experimenters.

### 4.6. Biochemical Indicator Testing

A fully automated biochemical analyzer (Shanghai Kehua Bio-engineering Co., Ltd., Shanghai, China) was used to detect the levels of fasting blood glucose (FBG), alanine transaminase (ALT), glutamic oxaloacetic transaminase (GOT/AST), triglycerides (TC), total cholesterol (TG), high-density lipoprotein cholesterol (HDL-C), and low-density lipoprotein cholesterol (LDL-C) in rat serum.

### 4.7. Serum Inflammatory Factor Levels and Fasting Insulin Tests

Fasting serum insulin (FINS), rat monocyte chemokine protein-1 (MCP-1), interleukin-6 (IL-6), and serum tumor necrosis factor alpha (TNFα) were all detected using the ELISA kit (LunChangShuo Biotechnology Co., Ltd., Beijing, China) in the rats. The kit and samples were removed 2 h before the operation to equilibrate the room temperature. The insulin resistance index (HOMA-IR) was calculated after calculating the content of each factor in the serum samples.

### 4.8. Tissue Embedding Sectioning and Staining

Firstly, the rats were anesthetized through an intraperitoneal injection of 3% pentobarbital sodium (30 mg/Kg), and their liver tissue was removed and fixed overnight (24 h) in 4% paraformaldehyde to ensure its fixation effect. Subsequently, paraffin embedding, dehydration, and sectioning were performed, and these liver tissue sections were stained with hematoxylin–eosin (HE) and observed under light microscope for pathologic changes in their liver histology. In addition, we placed fresh liver tissues in Optimal Cutting Temperature Compound (OCT) embedding agent, followed by cryosectioning, subjected the sections to Oil Red O staining, and, finally, sealed the sections with glycerol gelatin and observed the formation of lipid droplets in different groups of liver tissues under a light microscope. In addition to the above two staining methods, we also performed Sirius Red staining, which allowed us to clearly observe the distribution and deposition of collagen fibers in the liver, thus providing a basis for evaluating the extent and progression of liver fibrosis.

### 4.9. Data Analysis

The resulting data were analyzed in depth using GraphPad Prism 8.0 software to present the fluctuations in the data in the form of Mean ± Standard Deviation (Mean ± SD). In order to clarify the differences between the groups, statistical analyses were performed using one-way ANOVA or *t*-test.

### 4.10. Metabolomics

#### 4.10.1. Extraction of Metabolites

The samples were thawed at 4 °C. A 100 μL aliquot of each sample was then mixed with 100 μL of pre-cooled distilled water and 800 μL of a methanol/acetonitrile mixture (1:1, *v*/*v*), and thoroughly mixed. The samples were placed in an ice bath for 1 h of sonication. Subsequently, they were left at −20 °C for 2 h to promote protein precipitation. Next, the supernatant was centrifuged for 20 min (16,000× *g*, 4 °C). The supernatant was evaporated using a high-speed vacuum centrifuge. For mass spectrometry, 100 μL of a mixture of methanol and water (1:1, *v*/*v*) was added to the evaporated sample and centrifuged again for 20 min under the above conditions, and then the supernatant was removed and analyzed.

#### 4.10.2. LC-MS/MS Analysis

Chromatographic separation: Throughout the analysis, the samples were placed in an autosampler at 4 °C to maintain stability. A SHIMADZU-LC30 Ultra Performance Liquid Chromatography System (UPLC) was used with an ACQUITY UPLC^®^HSST3 (2.1 mm × 100 mm, 1.8 µm) (Waters, Milford, MA, USA) column. The injection volume was 4 μL, the column temperature was 40 °C, and the flow rate was 0.3 mL/min. The mobile phases were as follows: B: acetonitrile, A: 0.1% formic acid aqueous solution; the chromatographic gradient elution procedure was as follows: 0–2 min, 0B; 2–6 min, the B varied from 0% to 48%; 6–10 min, the B varied from 48% to 100%; 10–12 min, the B remained at 100%; 12–12.1 min, the B varied from 100% to 0%; 12.1–15 min, the B remained at 0%; 12.1–15 min, the B varied from 100% to 0%. From 12 to 12.1 min, B varied linearly from 100% to 0%, and from 12.1 to 15 min, B was maintained at 0%.

##### Mass Spectrometry Acquisition

Each sample was analyzed using electrospray ionization (ESI) in both positive (+) and negative (−) modes. The samples were separated via UPLC and then analyzed using mass spectrometry with a QE Plus mass spectrometer (Thermo Scientific, Waltham, MA, USA). Ionization was carried out using a HESI source with the following conditions: Spray voltage: 3.8 kV (+) and 3.2 kV (−); capillary temperature: 320 °C; sheath gas: 30 arbitrary units; aux gas: 5 arbitrary units; probe heater temperature: 350 °C; S-lens RF level: 50. The MS acquisition settings were as follows: acquisition time: 25 min; parent ion scan range: 80–1200 *m*/*z*; primary MS resolution: 70,000 @ *m*/*z* 200; AGC target: 3 × 10^6^; primary maximum IT: 100 ms. Secondary mass spectrometry analysis was conducted as follows: Triggered acquisition of secondary mass spectrometry (MS2scan) was performed on the 10 highest-intensity parent ions after each full scan. Secondary mass resolution was set to 17,500 @ *m*/*z* 200, with an AGC target of 1e5 and a maximum IT of 50 ms. MS2 activation was carried out using HCD with an isolation window of 2 *m*/*z* and normalized collision energies set to 20, 30, and 40.

#### 4.10.3. Data Preprocessing

Raw data were processed with MSDIAL software v4.90. for peak alignment, retention time correction, and peak area extraction. Metabolite identification involved precise mass number matching (mass tolerance < 10 ppm) and secondary spectral matching (mass tolerance < 0.01 Da) using databases such as HMDB, MassBank, and GNPS. Ion peaks with >50% missing values were excluded from the statistical analysis. Data from positive and negative ions were normalized by total peak area, integrated, and pattern recognition was performed using Python software (3.10), with preprocessing by Unit Variance Scaling (UV) for the subsequent analysis.

## 5. Conclusions

In summary, it was experimentally verified that BBD demonstrated significant therapeutic effects on high-fat chow-induced MASH rats and effectively improved hepatic lipid metabolism, fibrosis and inflammatory responses. Combining in-depth analysis of network pharmacology and metabolomics, we found that BBD mainly exerts therapeutic effects on MASH rats by regulating four metabolic biomarkers, such as Piceid, and five targets, such as ESR1, as well as involving two key compounds quercetin and 17-Beta-Estradiol, and mainly regulate the PI3K-AKT signaling pathway and bile secretion pathway to improve liver injury in MASH rats. We will follow up with further validation against relevant target pathways to provide new ideas for advancing the modernization of BBD research.

The whole study combined network pharmacological prediction, molecular docking, metabolomics and relevant experimental validation to systematically elucidate the mechanism of action of BBD on MASH, providing a multi-level chain of evidence; we also set up different drug concentration groups to elucidate the dose-dependent effect, which improved the credibility of the conclusions; moreover, the experiments used a high-fat-diet-induced MASH rat model, which mimicked the pathology of human disease characteristics, and the results are more translational. In the future, we will further combine with targeted metabolomics to deepen this mechanism.

## Figures and Tables

**Table 1:** The active ingredients of BBD.

Ingredient	Number	Bioactive Compound	Molecular Formula	Relative Molecular Mass	Key Effects
Ling Yang Jiao	LYJ1	Alanine	C_3_H_7_NO_2_	89.09 g/mol	Antipyretic, sedative, and liver-clearing
	LYJ2	Arginine	C_6_H_14_N_4_O_2_	174.2 g/mol	
	LYJ3	Aspartic acid	C_4_H_7_NO_4_	133.1 g/mol	
	LYJ4	Cysteine	C_3_H_7_NO_2_S	121.16 g/mol	
	LYJ5	Glutamic acid	C_5_H_9_NO_4_	147.13 g/mol	
	LYJ6	Histidine	C_6_H_9_N_3_O_2_	155.15 g/mol	
	LYJ7	Isoleucine	C_6_H_13_NO_2_	131.17 g/mol	
	LYJ8	Leucine	C_6_H_13_NO_2_	131.17 g/mol	
	LYJ9	L-Valine	C_5_H_11_NO_2_	117.15 g/mol	
	LYJ10	Methionine	C_5_H_11_NO_2_S	149.21 g/mol	
	LYJ11	Phenylalanine	C_9_H_11_NO_2_	165.19 g/mol	
	LYJ12	Proline	C_5_H_9_NO_2_	115.13 g/mol	
	LYJ13	Tryptophan	C_11_H_12_N_2_O_2_	204.22 g/mol	
	LYJ14	Tyrosine	C_9_H_11_NO_3_	181.19 g/mol	
	LYJ15	Threonine	C_4_H_9_NO_3_	119.12 g/mol	
Niu Huang	NH1	12α-Trihydroxy-5β-cholestane-24-oic acid methyl ester	C_25_H_42_O_5_	422.6 g/mol	Antispasmodic and neuroprotective
	NH2	Deoxycholic acid methyl ester	C_25_H_42_O_4_	406.6 g/mol	
	NH3	Deoxycholic acid	C_24_H_40_O_4_	392.6 g/mol	
	NH4	ZINC01280365	C_21_H_30_O_3_	330.5 g/mol	
	NH5	Cholesterol	C_27_H_46_O	386.7 g/mol	
San Qi	SQ1	Glycyrrhizin	C_15_H_12_O_4_	256.25 g/mol	Hemostatic and anti-fibrotic
	SQ2	Quercetin	C_15_H_10_O_7_	302.23 g/mol	
	SQ3	Diisooctyl phthalate	C_24_H_38_O_4_	390.6 g/mol	
	SQ4	β-Sitosterol	C_29_H_50_O	414.7 g/mol	
	SQ5	Soy sterols	C_29_H_48_O	412.7 g/mol	
	SQ6	Ethyl linoleate	C_20_H_36_O_2_	308.5 g/mol	
She Dan	SD1	Bile acids	C_24_H_40_O_5_	408.6 g/mol	Hepatoprotective and antimicrobial
	SD2	Glycocholic acid	C_26_H_43_NO_6_	465.6 g/mol	
	SD3	α-Usodeoxycholic acid	C_24_H_40_O_6_	424.6 g/mol	
	SD4	Taurocholic acid	C_26_H_45_NO_7_S	515.7 g/mol	
She Xiang	SX1	Androst-4-ene-3,17-dione	C_19_H_26_O_2_	286.4 g/mol	Neurostimulant and cardioprotective
	SX2	Testosterone	C_19_H_28_O_2_	288.4 g/mol	
	SX3	3,5-Dihydroxybenzoic acid	C_7_H_6_O_4_	154.12 g/mol	
	SX4	Musenin	C_51_H_82_O_21_	1031.2 g/mol	
	SX5	17β-Estradiol	C_18_H_24_O_2_	272.4 g/mol	
	SX6	Allantoin	C_4_H_6_N_4_O_3_	158.12 g/mol	
	SX7	3-Methylcyclotridecan-1-one	C_14_H_26_O	210.36 g/mol	
	SX8	Musk pyridine	C_16_H_25_N	231.38 g/mol	
	SX9	SCHEMBL2197370	C_15_H_26_O	222.37 g/mol	
	SX10	Decylamine	C_30_H_40_C_l2_N_4_	527.6 g/mol	
	SX11	Musk lactone A1	C_8_H_18_O_5_S	226.29 g/mol	
	SX12	Methylpiperonylphenol	C_10_H_12_O	148.2 g/mol	
	SX13	Cyclotetradecan-1-one	C_14_H_26_O	210.36 g/mol	
	SX14	3β-hydroxy-5α-androstan-17-one	C_19_H_30_O_2_	290.4 g/mol	
	SX15	α-Estradiol	C_18_H_24_O_2_	272.4 g/mol	
	SX16	Androstenone	C_19_H_30_O_2_	290.4 g/mol	
	SX17	Cyclopentadienone	C_26_H_46_N_2_O	402.7 g/mol	
	SX18	Cholesterol	C_27_H_46_O	386.7 g/mol	
	SX19	Musk ketone	C_16_H_30_O	238.41 g/mol	
	SX20	2,6-decamethylene pyridine	C_15_H_23_N	217.35 g/mol	
	SX21	3α-hydroxy-5α-androstan-17-one	C_19_H_30_O_2_	290.4 g/mol	
	SX22	Diethyltoluamide	C_12_H_17_NO	191.27 g/mol	
	SX23	Musk ketone	C_15_H_28_O	224.38 g/mol	
	SX24	2,6-Ninomethylidenepyridine	C_14_H_21_N	203.32 g/mol	
	SX25	5-cis-cyclotetradecen-1-one	C_14_H_24_O	208.34 g/mol	
Zhen Zhu	ZZ1	Aluminum	Al	26.981 g/mol	Sedative and corneal repair
	ZZ2	Copper	Cu	63.55 g/mol	
	ZZ3	Iron	Fe	55.84 g/mol	
	ZZ4	Silicon	Si	28.085 g/mol	

‘Ingredient’ refers to the six main active ingredients of BBD, and ‘Number’ refers to the bioactive compounds corresponding to each of the six main active ingredients.

**Table 2:** The top 10 core targets screened by Centiscape2.2.

Betweenness unDir	Closeness unDir	Degree unDir	Name
1301.063994	0.005291005	250	INS
827.1577515	0.005076142	234	TNF
648.0910967	0.005050505	234	AKT1
724.0959497	0.005076142	234	IL6
763.3984781	0.005000000	230	TP53
682.0782369	0.004878049	220	PPARG
482.4419534	0.004854369	216	IL1B
595.3456063	0.004784689	214	ESR1
542.9045489	0.004739336	210	STAT3
325.8217509	0.004739336	208	JUN

**Table 3:** The binding energy between the top 10 core targets and their corresponding components.

Protein	Ligand	Binding Energy (kcal/mol)
INS	quercetin	−6.6
TNF	quercetin	−6.8
AKT1	quercetin	−6.3
IL6	17-beta-estradiol	−7.2
TP53	17-beta-estradiol	−7.7
PPARG	quercetin	−7.1
IL1B	DFV	−7.0
ESR1	quercetin	−7.3
STAT3	17-beta-estradiol	−7.4
JUN	glycocholic acid	−5.6

**Table 4:** OPLS-DA model evaluation parameters.

Comparison Group	R2X (cum)	R2Y (cum)	Q2 (cum)	RMSEE
A vs. B	0.316	0.99	0.897	0.0543
B vs. D	0.291	0.966	0.529	0.102

## Data Availability

Data presented in this study is contained within the article and supplementary material. Further inquiries can be directed to the corresponding author.

## Supplementary Materials

The following supporting information can be downloaded at: https://www.mdpi.com/article/10.3390/ph18081111/s1, File S1: BBD chemical fingerprint (HPLC).

## Abbreviations

ALT	Alanine transaminase
BATMAN-TCM	Bioinformatics Analysis Tool for Molecular Mechanism of Traditional Chinese Medicine
BBD	Babao Dan
CYP27A1	Sterol 27-hydroxylase
CYP7A1	Cholesterol 7α-hydroxylase
CYP8B1	Sterol 12α-hydroxylase
DAVID	Database for Annotation, Visualization and Integrated Discovery
DisGeNet	Database of gene–disease associations
DL	Drug likeness
ESR1	Estrogen receptor 1
FBG	Fasting blood glucose
FINS	Fasting serum insulin
GO	Gene Ontology
GOT/AST	Glutamic oxaloacetic transaminase
GSKL	GanShuangKeLi
HDL-C	High-density lipoprotein cholesterol
HE	Hematoxylin–eosin
IL-6	Interleukin-6
KEGG	Kyoto Encyclopedia of Genes and Genomes
LDL-C	Low-density lipoprotein cholesterol
MCP-1	Rat monocyte chemokine protein-1
NASH	Nonalcoholic steatohepatitis
MASH	Metabolic dysfunction-associated steatohepatitis
MAFLD	Metabolic-associated fatty liver disease
OB	Oral bioavailability
OMIM	Online Mendelian Inheritance in Man
PPARG	Peroxisome proliferator-activated receptor gamma
PPI	Protein–protein interaction
STAT3	Signal Transducer and Activator of Transcription 3
TC	Triglyceride
TCMSP	Traditional Chinese Medicine Systems Pharmacology Database and Analysis Platform
TG	Total cholesterol
TNFα	Serum tumor necrosis factor alpha
TP53	Tumor protein p53
TTD	Therapeutic Target Database
