# Supplementary material for: UPLC-MS/MS Metabolomics Reveals Babao Dan’s Mechanisms in MASH Treatment with Integrating Network Pharmacology and Molecular Docking
Source: Pharmaceuticals (Basel). 2025 Jul 25;18(8):1111. doi: 10.3390/ph18081111 (PMC12388948; doi:10.3390/ph18081111)

The high-performance liquid chromatograph was an Agilent 1100 series, including: G1379A online deaerator, G1311A quaternary pump, G1313A automatic sampler, G1316A temperature control box, G1315B diode array detector, and Agilent chemical workstation (Rev A.10.02. [1757]).

The chromatographic column was Hadesil C18-K column (4.6 mm × 250 mm, 5.0 μm), the mobile phase was acetonitrile water 0.1% TFA system, and the gradient elution procedure was as follows: 0 min, acetonitrile water 0.1% TFA = 10:80:10, 0–60 min, acetonitrile 10% → 80%, water 80% → 10%, 0.1% TFA maintained at 10%. The flow rate was 0.8 mL/min and the column temperature was 40℃. The detection wavelength was 230 nm.

1 capsule (0.3 g) of BaBaoDan was taken into a beaker, 100 mL of purified water was added, then the mixture was stirred and heated to boil for 30 minutes, then cooled down. The volume of the mixture was replenished to 100 mL with purified water. 1 mL of the mixture was taken into a centrifuge tube, and was centrifuged at 12000 rpm for 10 minutes, then the supernatant was transferred into the sample bottle, and 1 μL was injected for detection.

The chromatogram was as follows:

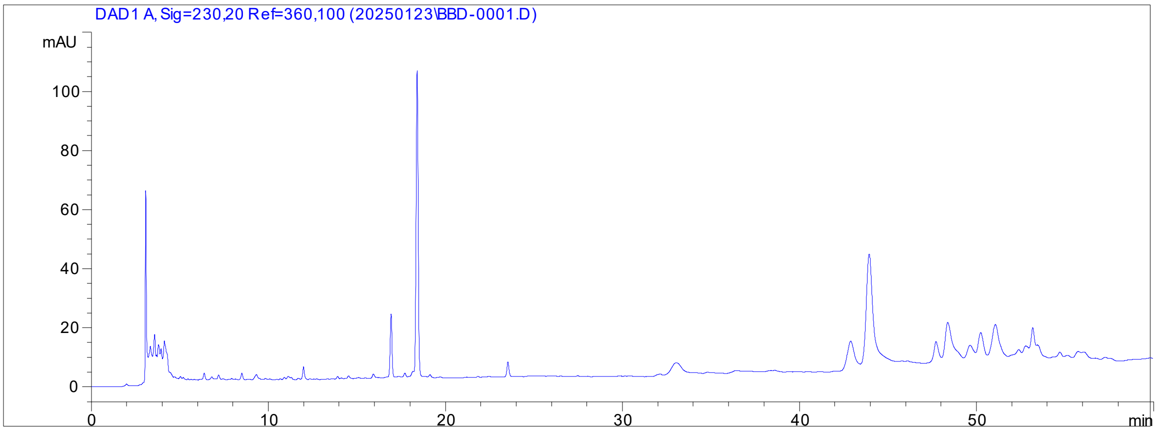

Supplement: Supplementary file 1 [file pharmaceuticals-18-01111-s001.zip › Supplementary File S1.pdf]
